# Supplementary material for: Proteomic Analysis Revealed Different Molecular Mechanisms of Response to PEG Stress in Drought-Sensitive and Drought-Resistant Sorghums
Source: Int J Mol Sci. 2022 Oct 31;23(21):13297. doi: 10.3390/ijms232113297 (PMC9656428; doi:10.3390/ijms232113297)
Supplement: Supplementary file 1 [file ijms-23-13297-s001.zip › Supplementary Figures.pdf]

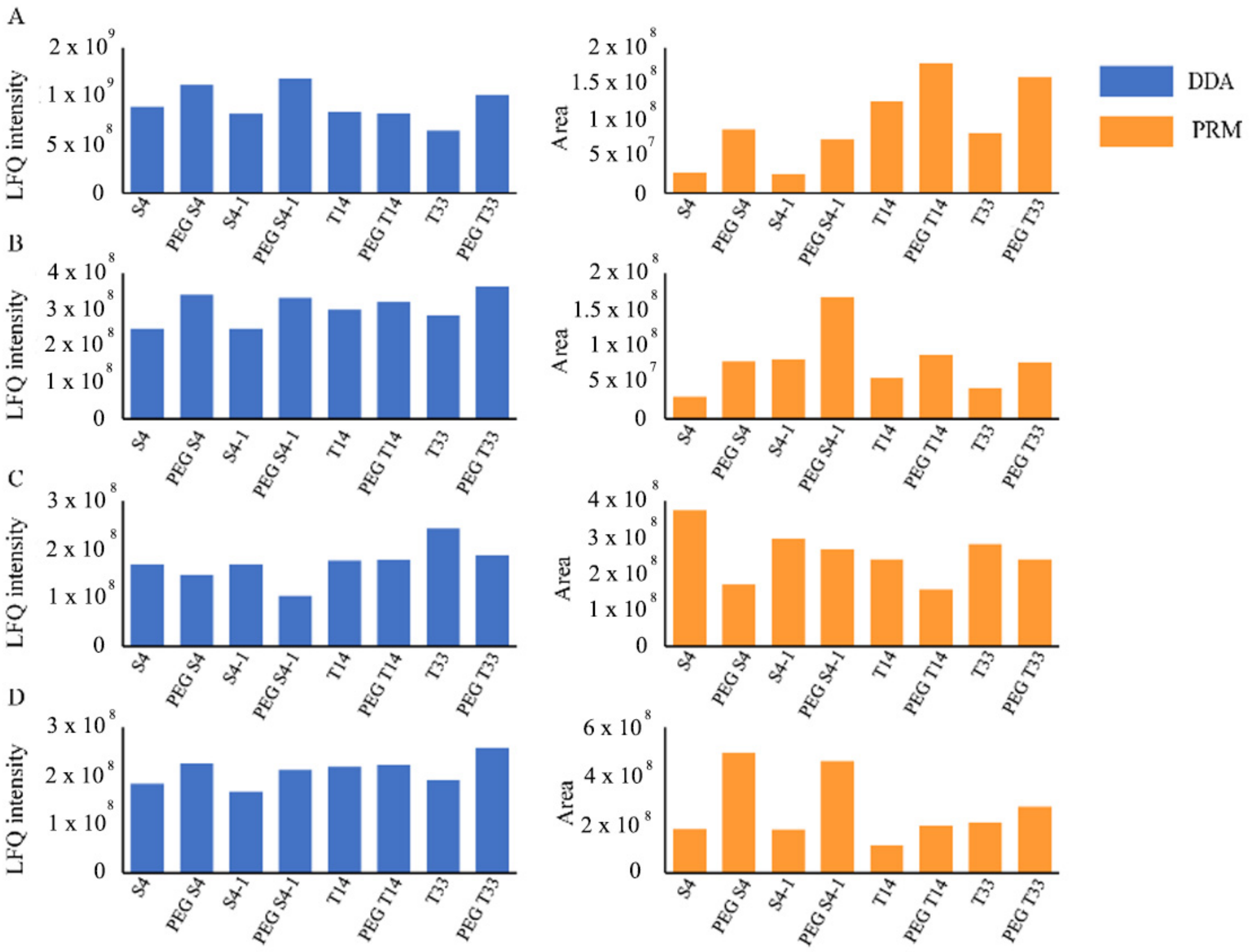

Figure S1. PRM verification of four proteins related to PEG treatment. (**A**) HATPase_c domain-containing protein (A0A194YLP4). (**B**) Uncharacterized protein (C5WVD3). (**C**) 3-isopropylmalate dehydratase (C5XT62). (**D**) Ribonuclease (C5XTA6). Left was the result of the proteome and right was PRM verification.

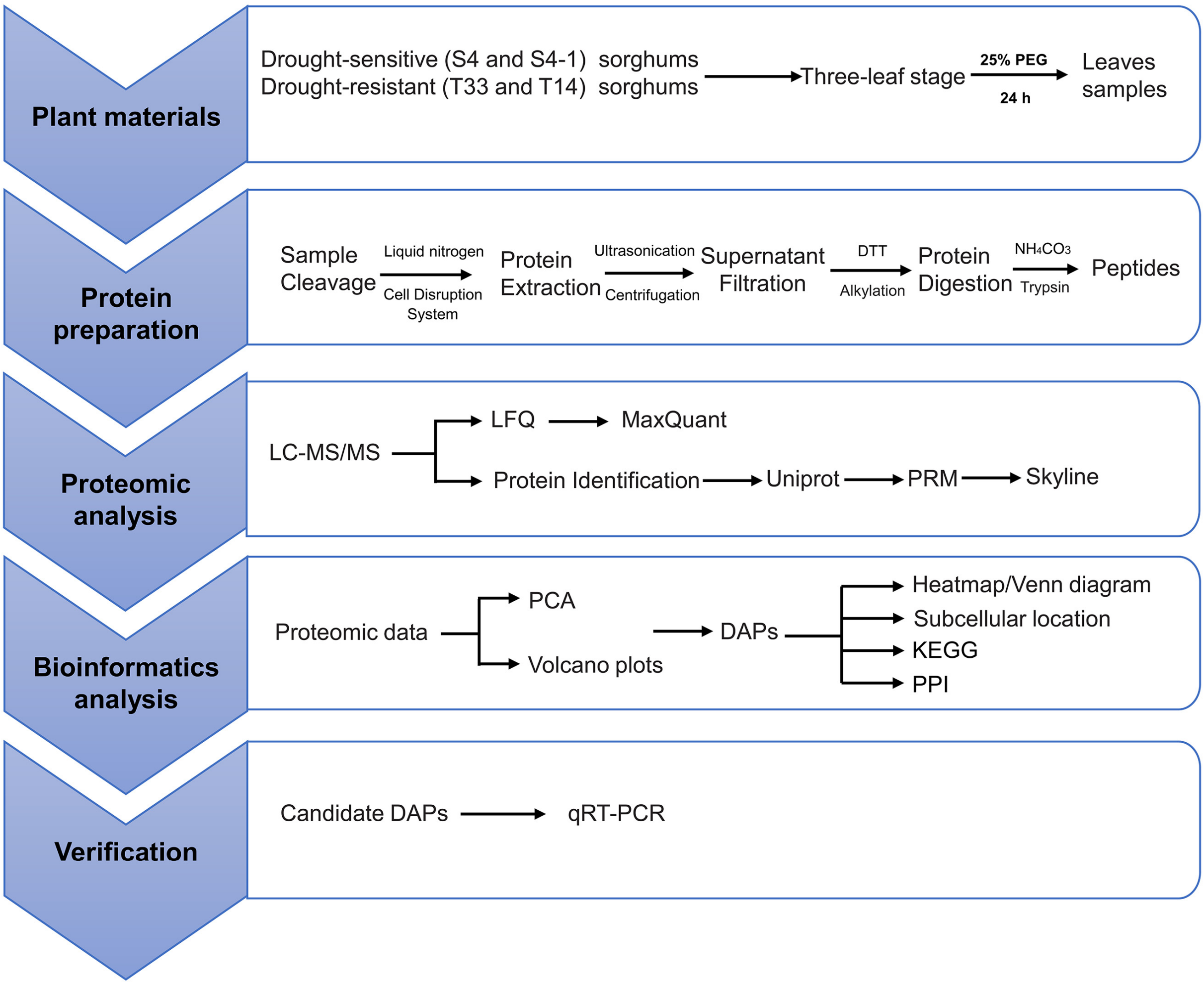

Figure S2. The flowchart of proteomics analysis of the drought-resistant (T14 and T33) and drought-sensitive (S4 and S4-1) sorghums.
